# Subphenotypes of inflammatory bowel disease are characterized by specific serum protein profiles

**DOI:** 10.1371/journal.pone.0186142

**Published:** 2017-10-05

**Authors:** Erik Andersson, Daniel Bergemalm, Robert Kruse, Gunter Neumann, Mauro D’Amato, Dirk Repsilber, Jonas Halfvarson

**Affiliations:** 1 School of Medical Sciences, Faculty of Medicine and Health, Örebro University, Örebro, Sweden; 2 Department of Gastroenterology, Faculty of Medicine and Health, Örebro University, Örebro, Sweden; 3 Clinical Epidemiology Unit, Department of Medicine Solna, Karolinska Institutet, Stockholm, Sweden; 4 BioDonostia Health Research Institute, San Sebastian, Spain; 5 IKERBASQUE Basque Foundation for Science, Bilbao, Spain; Duke University, UNITED STATES

## Abstract

**Objective:**

Genetic and immunological data indicate that inflammatory bowel disease (IBD) are characterized by specific inflammatory protein profiles. However, the serum proteome of IBD is still to be defined. We aimed to characterize the inflammatory serum protein profiles of Crohn’s disease (CD) and ulcerative colitis (UC), using the novel proximity extension assay.

**Methods:**

A panel of 91 inflammatory proteins were quantified in a discovery cohort of CD (n = 54), UC patients (n = 54), and healthy controls (HCs; n = 54). We performed univariate analyses by t-test, with false discovery rate correction. A sparse partial least-squares (sPLS) approach was used to identify additional discriminative proteins. The results were validated in a replication cohort.

**Results:**

By univariate analysis, 17 proteins were identified with significantly different abundances in CD and HCs, and 12 when comparing UC and HCs. Additionally, 64 and 45 discriminant candidate proteins, respectively, were identified with the multivariate approach. Correspondingly, significant cross-validation error rates of 0.12 and 0.19 were observed in the discovery cohort. Only FGF-19 was identified from univariate comparisons of CD and UC, but 37 additional discriminant candidates were identified using the multivariate approach. The observed cross-validation error rate for CD *vs*. UC remained significant when restricting the analyses to patients in clinical remission. Using univariate comparisons, 16 of 17 CD-associated proteins and 8 of 12 UC-associated proteins were validated in the replication cohort. The area under the curve for CD and UC was 0.96 and 0.92, respectively, when the sPLS model from the discovery cohort was applied to the replication cohort.

**Conclusions:**

By using the novel PEA method and a panel of inflammatory proteins, we identified proteins with significantly different quantities in CD patients and UC patients compared to HCs. Our data highlight the potential of the serum IBD proteome as a source for identification of future diagnostic biomarkers.

## Introduction

Inflammatory bowel disease (IBD), comprising Crohn’s disease (CD) and ulcerative colitis (UC), is a chronic inflammatory disease affecting the gastrointestinal tract. The inflammation arises at the intersection of genetic predisposition and factors related to the exposome. Current theories suggest that the disease is caused by an aberrant immune response to commensal gut microbiota in genetically predisposed individuals, which is precipitated by environmental factors.[1] However, the exact etiology of inflammatory bowel disease (IBD) remains unknown.

There has been a great progress within the field of genetics, genome wide association studies (GWAS) and subsequent meta-analyses have contributed significantly to our understanding of the genetic landscape of IBD. More than 200 genetic variants have been associated with the disease, but the causal gene has only been identified in a subset of theses variants. As a consequence, the impact of IBD on the proteome remains largely undescribed.

The majority of identified genetic variants represent pathways of innate and adaptive immunity.[2] The key role of the immune response is also supported by data generated in mice models, in vitro and in vivo studies. However, the characteristics of both the innate immune response and adaptive immune response differ between CD and UC.[3, 4] Pronounced differences in inflammatory mediators such as cytokines and chemokines have also been shown between patients with CD and UC.[1]

Recently, the categorization of IBD into CD and UC has been suggested to be overly simplistic.[5] Some genetic variants are disease-specific and the genetic risk scores, generated from all known risk alleles for IBD, seem to be associated with different subphenotypes of CD, such as ileal CD and colonic CD, as well as UC.[6]

Thus, based on genetic variants and immunological data it can be hypothesized that IBD, and possibly also subphenotypes of the disease, might be characterized by specific inflammatory profiles within the serum proteome.

There is considerable variation within individuals in the concentration of different proteins in serum, which makes the use of traditional proteomic techniques such as 2-dimensional gel electrophoresis (2DE) and mass spectrometry, challenging.[7] In contrast, antibody-based techniques such as proximity extension assay (PEA) offer a combination of high sensitivity and a wide quantitative window of individual proteins, which avoids the problems associated with variation in protein concentration.[8] We aimed to characterize the inflammatory serum protein profiles of CD and UC using the novel PEA technique.

## Materials and methods

### Population

Adult patients with CD and UC were consecutively recruited at the outpatient IBD clinic of Örebro University Hospital, Sweden. Similarly, consecutive healthy blood donors with no history of chronic gastrointestinal disease were recruited at Örebro University Hospital. All individuals were recruited between 2005 and 2012 and the cohort has been described previously in detail.[9] After obtaining an informed written consent, blood samples were collected and the serum was separated after centrifugation at 2,400 *g* for six minutes at room temperature. All serum samples were stored as aliquots at −80°C. The diagnoses of CD and UC were based on clinical, endoscopic, radiological, and histological criteria.[10] Disease characteristics were classified according to the Montreal classification,[11] with the exception of disease activity, for which the physician’s global assessment was used.[12, 13] A random sample of CD (n = 54) were selected, as well as UC patients (n = 54) and healthy blood donors with no history of chronic gastrointestinal disease (HC; n = 54), both groups were matched according to sex and age (± 5 years). Altogether, these 162 individuals constituted the discovery cohort. For validation of results obtained from the discovery cohort, a replication cohort consisting of 30 CD patients, 30 UC patients, and 30 HCs was recruited in a similar manner with matching according to sex and age (± 5 years).

The study was approved by the Örebro University Hospital ethics committee.

### Protein analysis

The commercially available panel, ProSeek Multiplex Inflammation I 96x96 (Olink Proteomics, Uppsala, Sweden) consists of 91 preselected proteins, all releated to inflammation (S1 Table). The concentrations of the proteins in the panel were assessed as previously described.[8] Briefly, a PEA was performed, where pairs of antibodies with oligonucleotides attached were incubated with the antigens. Oligonucleotides in close proximity produced a template for hybridization and extension. Pre-amplification was based on universal primers and PCR. Residual primers were digested before quantification with specific primers on a quantitative real-time PCR chip (Dynamic Array IFC; Fluidigm Biomark) on a Biomark HD Instrument. The analyses were performed at the Clinical Biomarkers Facility, Science for Life Laboratory, Uppsala. Normalized log_2_ values corresponding to protein quantities were generated with the Olink Wizard for GenEx (Multid Analyses, Sweden).

### Statistics

Continuous variables, representing clinical characteristics, are presented as median and interquartile range (IQR) and differences were tested with the Mann-Whitney U test. Corresponding categorical data are presented as frequencies, and they were compared using Pearson Chi-square test or Fisher’s exact test when appropriate.

Proteins with signals below the LOD in > 80% of the samples were excluded, if the remaining signals were evenly distributed between cases and controls. This was done in order to reduce the effect of biologically irrelevant differences or non-informative protein features. All samples in the discovery cohort were normalized using quantile normalization, and the concentration of proteins below the LOD was then reset to zero.

In order to identify possible outliers, and to evaluate consistency of the data in the discovery cohort, principal component analyses (PCAs) were performed and score plots were visually inspected.

Univariate analyses were performed by Welsh t-test. P-values were adjusted for multiple comparisons using a false discovery rate (FDR) approach with q-values reported. Proteins were regarded as being differentially regulated in the discovery cohort if they had a fold change of at least 1.2 and a q-value of < 0.05.

Each sample in the replication cohort was normalized independently against all samples of the discovery cohort, to simulate diagnostic conditions for newly measured samples. Proteins showing significant up- or down-regulation, based on univariate analysis of the discovery cohort, were considered to be validated if they had a similar (defined as the 95% confidence interval of fold change for each protein in the discovery cohort) or larger fold change of identical direction in the replication cohort.

Multivariate analysis comprised both principle component analysis (PCA) and sparse partial least-squares analysis (sPLS).[14] Being a supervised learning method, the sPLS model is optimized to separate groups. For the sPLS analysis, CD patients were stratified based on disease location. Due to the complexity of the model and the limited number of patients, disease location was divided into two categories: colonic disease (L2) and ileal/ileocolonic disease (L1/L3). A second series of analyses was performed stratifying for clinical disease activity and excluding patients with active disease. Variable importance in the projection (VIP) was calculated for all variables and the analysis was optimized for both the number of variables and the number of components to use in the prediction model, with a rigorous double cross-validation design.[15, 16] The selection of proteins was based on the optimized cut-off for the VIP scores. The prediction model was validated through leave-one-out (LOO) cross-validation and then tested on the replication cohort data set to determine its accuracy in class prediction of disease groups (UC, colonic CD, ileal/ileocolonic CD, CD in clinical remission and UC in clinical remission).[17] Significance of the observed LOO error rates was established by resampling analysis, i.e. randomly permuting the class labels and re-running the double cross-validation analyses, to be able to calculate permutation p-values for the observed LOO prediction hit rates for the original data.

Statistical analyses and data processing were performed in R version 3.2.2 (R Foundation for Statistical Computing, Vienna, Austria) with the packages qvalue 2.0.0 (John Storey, 2015) and mix0mics 5.2.0 (Kim-Anh Le Cao, 2015), and IBM SPSS Statistics for Windows 22.0 (IBM Corp., Armonk, NY).

## Results

### Clinical and demographic characteristics of the discovery cohort

Clinical characteristics of patients with CD and UC in the discovery cohort are given in Table 1. The median (IQR) disease duration at inclusion in CD and UC patients was 17.5 (8–28) and 13 (5–25) years, respectively.

**Table 1:** Clinical and demographic characteristics of the discovery cohort and the replication cohort at inclusion.

	Discovery cohort		Replication cohort	
	Crohn’s disease	Ulcerative colitis	Crohn’s disease	Ulcerative colitis
	n = 54	n = 54	n = 30	n = 30
**Male sex**	36 (66.7%)	36 (66.7%)	20 (66.7%)	20 (66.7%)
**Median (range) age at diagnosis, years**	28 (18.9–37.1)	30 (19.5–40.5)	25.0 (15.9–34.2)	26.0 (20.1–31.9)
**Disease location**				
Ileal (L1)	15 (27.8%)		6 (20.0%)	
Colonic (L2)	13 (24.1%)		14 (46.7%)	
Ileocolonic (L3)	22 (40.7%)		10 (33.3%)	
Upper disease (L4)	4 (7.4%)		0	
**Disease behavior**				
Non-stricturing, non-penetrating (B1)	22 (40.7%)		16 (53.3%)	
Stricturing (B2)	21 (38.9%)		11 (36.7%)	
Penetrating (B3)	11 (20.4%)		3 (10.0%)	
Perianal fistulas	6 (11.1%)		7 (23.3%)	
**Extent of disease**				
Proctitis (E1)		7 (13.0%)		7 (23.3%)
Left-sided colitis (E2)		25 (46.3%)		8 (26.7%)
Extensive colitis (E3)		22 (40.7%)		15 (50.0%)
**Clinical disease activity**^a^				
Remission	37 (68.5%)	42 (77.8%)	24 (80.0%)	22 (73.3%)
Active	16 (29.6%)	12 (22.2%)	6 (20.0%)	8 (26.7%)
**Medications**^b^				
5ASA/SASP	9 (16.7%)	28 (51.9%)	4 (13.3%)	13 (43.3%)
Corticosteroids	9 (16.7%)	8 (14.8%)	3 (10.0%)	8 (26.7%)
Thiopurines	14 (25.9%)	12 (22.2%)	8 (26.7%)	4 (13.3%)
Methotrexate	2 (3.7%)	2 (3.7%)	3 (10.0%)	1 (3.3%)
Anti-TNF	1 (1.9%)	0	3 (10.0%)	0
No drugs	23 (42.6%)	13 (24.1%)	14 (46.7%)	12 (40.0%)
**Previous surgical resection**	35 (64.8%)	6 (11.1%)	16 (53.3%)	3 (10.0%)

^a^ Data on disease activity were not available in one patient with Crohn’s disease in the discovery cohort.

^b^ Some patients were in a combination of different treatments (discovery cohort: CD, n = 4; UC, n = 10; replication cohort: CD, n = 5; UC, n = 7).

### Preparation of data set

Serum samples from 162 individuals in the discovery cohort were run in parallel on two ProSeek plates. Ninety-one target proteins were quantified successfully. However, IL-13, IL-33, IL-1 alpha, and TSLP were below LOD in > 80% of the individuals. These proteins were therefore excluded from further analyses, since the concentrations observed in the remaining samples were evenly distributed between CD patients, UC patients, and HCs.

### Differentially regulated proteins in the discovery cohort identified by univariate analysis

**Fig 1:**
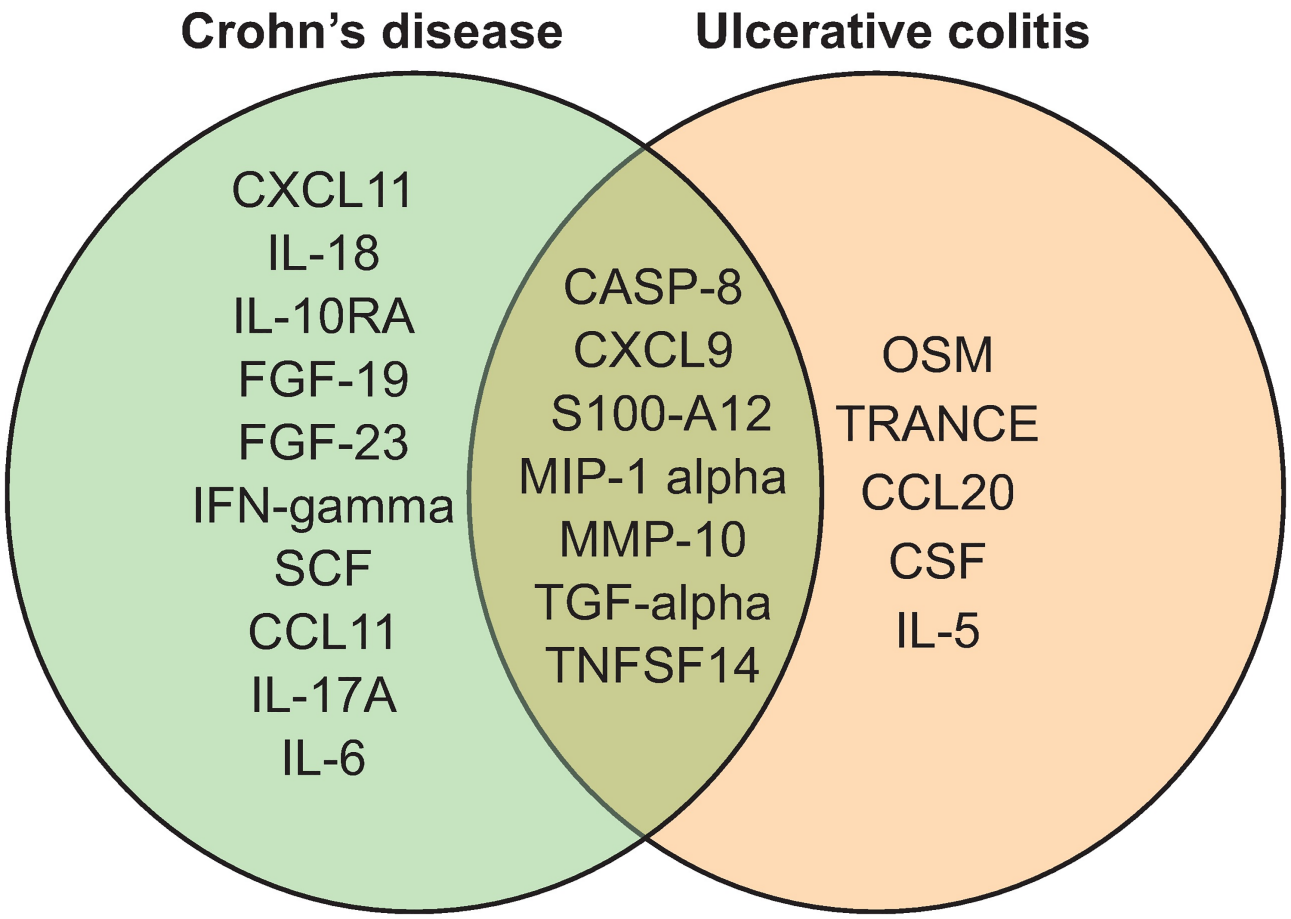
Venn diagram of significantly altered proteins (q < 0.05, fold change > 20%) from comparison of UC patients vs. healthy controls (HCs) and CD patients vs. HCs in the discovery cohort. Seven markers were common between the analyses.

Differentially regulated proteins between different groups of individuals, identified by univariate analysis, are shown in Table 2. Twenty-two proteins were identified by univariate analysis when CD and UC patients were compared to HCs. Seven of these 22 proteins differed in both CD and UC (Fig 1).

**Table 2:** Differentially regulated proteins in Crohn’ disease patients, ulcerative colitis patients, and healthy controls (from univariate analysis).

Protein	Symbol	Uniprot ID	Fold change (CI 95%)	q-value
***Crohn's disease vs*. *healthy controls***				
**Fibroblast growth factor 19**	FGF-19	O95750	0.42 (0.31–0.59)	< 0.001
**Macrophage inflammatory protein 1-alpha**	MIP-1 alpha	P10147	0.58 (0.39–0.88)	0.03
**Transforming growth factor alpha**	TGF-alpha	P01135	0.68 (0.59–0.79)	< 0.001
**Tumor necrosis factor ligand superfamily member 14**	TNFSF14	O43557	0.80 (0.69–0.91)	0.008
**Stem cell factor**	SCF	P21583	0.82 (0.72–0.93)	0.01
**Matrix metalloproteinase-10**	MMP-10	P09238	1.22 (1.03–1.46)	0.04
**Fibroblast growth factor 23**	FGF-23	Q9GZV9	1.22 (1.03–1.45)	0.04
**Eotaxin-1**	CCL11	P51671	1.23 (1.11–1.36)	0.001
**Interferon gamma**	IFN-gamma	P01579	1.23 (1.03–1.47)	0.04
**Interleukin-18**	IL-18	Q14116	1.25 (1.09–1.43)	0.009
**Interleukin-17A**	IL-17A	Q16552	1.25 (1.08–1.45)	0.01
**Protein S100-A12**	EN-RAGE	P80511	1.31 (1.06–1.62)	0.03
**Interleukin-10 receptor subunit alpha**	IL-10RA	Q13651	1.32 (1.10–1.58)	0.01
**C-X-C motif chemokine 11**	CXCL11	O14625	1.34 (1.11–1.62)	0.01
**Caspase 8**	CASP-8	Q14790	1.36 (1.17–1.59)	0.001
**C-X-C motif chemokine 9**	CXCL9	Q07325	1.41 (1.16–1.72)	0.007
**Interleukin-6**	IL-6	P05231	1.42 (1.07–1.89)	0.03
***Ulcerative colitis vs*. *healthy controls***				
**Macrophage inflammatory protein 1-alpha**	MIP-1 alpha	P10147	0.56 (0.37–0.85)	0.02
**Transforming growth factor alpha**	TGF-alpha	P01135	0.70 (0.60–0.81)	< 0.001
**C-C motif chemokine 20**	CCL20	P78556	0.70 (0.56–0.88)	0.01
**Oncostatin-M**	OSM	P13725	0.77 (0.64–0.93)	0.02
**Tumor necrosis factor ligand superfamily member 14**	TNFSF14	O43557	0.78 (0.69–0.88)	0.002
**TNF-related activation-induced cytokine**	TRANCE	O14788	0.81 (0.68–0.97)	0.04
**Stem cell factor**	SCF	P21583	0.82 (0.72–0.95)	0.02
**Caspase 8**	CASP-8	Q14790	1.21 (1.06–1.39)	0.02
**C-X-C motif chemokine 9**	CXCL9	Q07325	1.25 (1.04–1.50)	0.03
**Matrix metalloproteinase-10**	MMP-10	P09238	1.29 (1.07–1.55)	0.02
**Protein S100-A12**	EN-RAGE	P80511	1.37 (1.13–1.65)	0.01
**Interleukin-5**	IL-5	P05113	1.50 (1.11–2.03)	0.02
***Crohn's disease vs*. *ulcerative colitis***				
**Fibroblast growth factor 19**	FGF-19	O95750	0.51 (0.36–0.70)	0.007
***Ileal/ileocolonic Crohn’s disease vs*. *ulcerative colitis***				
**Fibroblast growth factor 19**	FGF-19	O95750	0.43 (0.29–0.64)	0.003
***Colonic Crohn’s disease vs*. *ulcerative colitis***				
**C-C motif chemokine 28**	CCL28	Q9NRJ3	0.75 (0.64–0.87)	0.04

Only fibroblast growth factor 19 (FGF-19) was differentially regulated when comparing CD patients and UC patients. Particularly, CD patients who had undergone any previous surgical resection had lower abundance of FGF19 (84.89, 95% CI 59.79–120.52 *vs*. 243.48, 95% CI 174.39–339.93 respectively, q-value = 0.004). No proteins were identified by univariate analysis as being significantly altered in ileal/ileocolonic CD (L1/L3) relative to colonic CD (L2).

### Differentially regulated proteins in the discovery cohort identified by multivariate analysis

**Fig 2:**
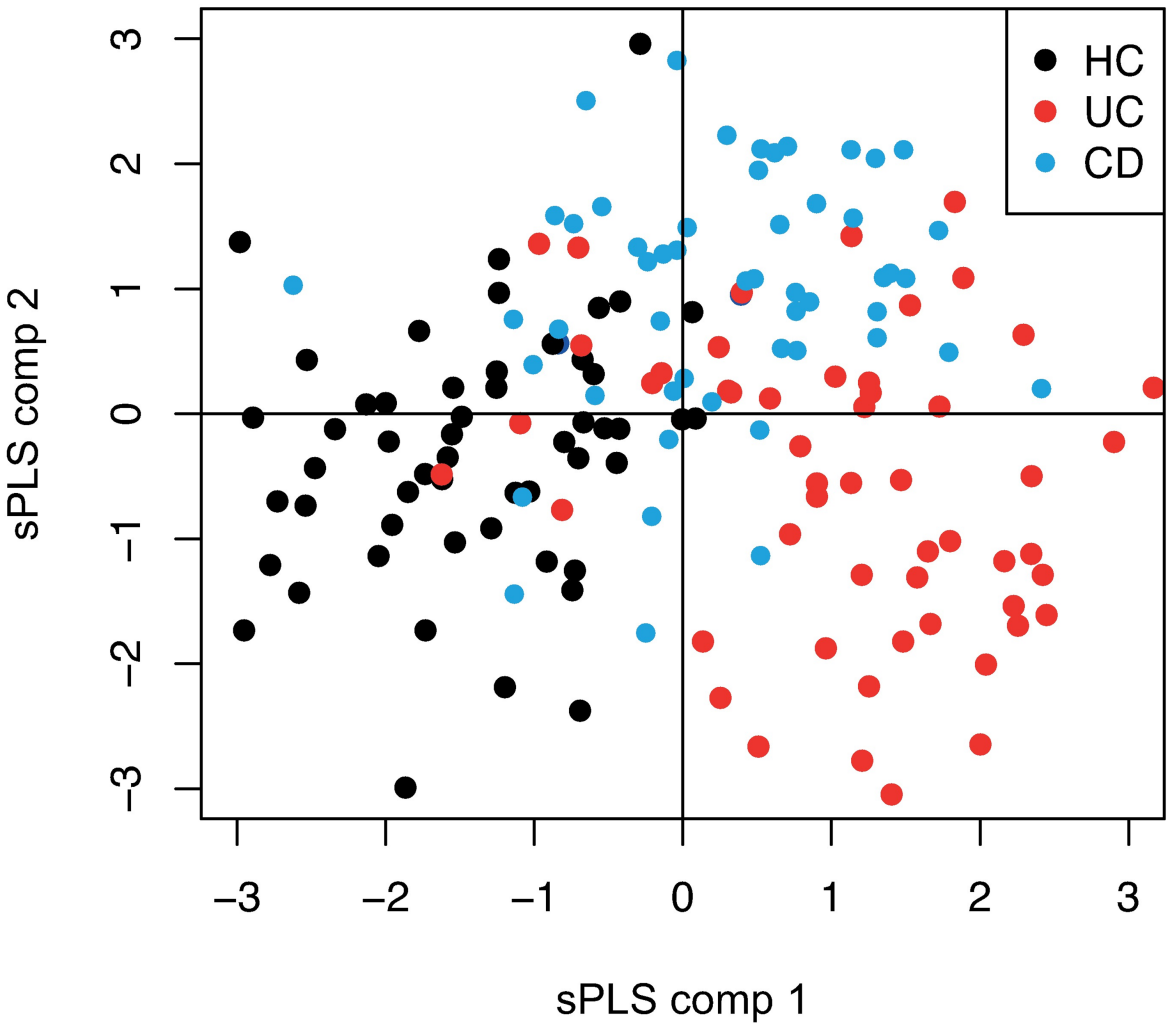
sPLS plot showing the distribution of all samples from CD patients, UC patients, and healthy controls (HCs) in the discovery cohort, based on the 20 most important proteins from the multivariate analysis.

The score plots for the first two components of the sPLS model showed a partial separation of CD, UC, and HC samples within the discovery cohort (Fig 2).

**Fig 3:**
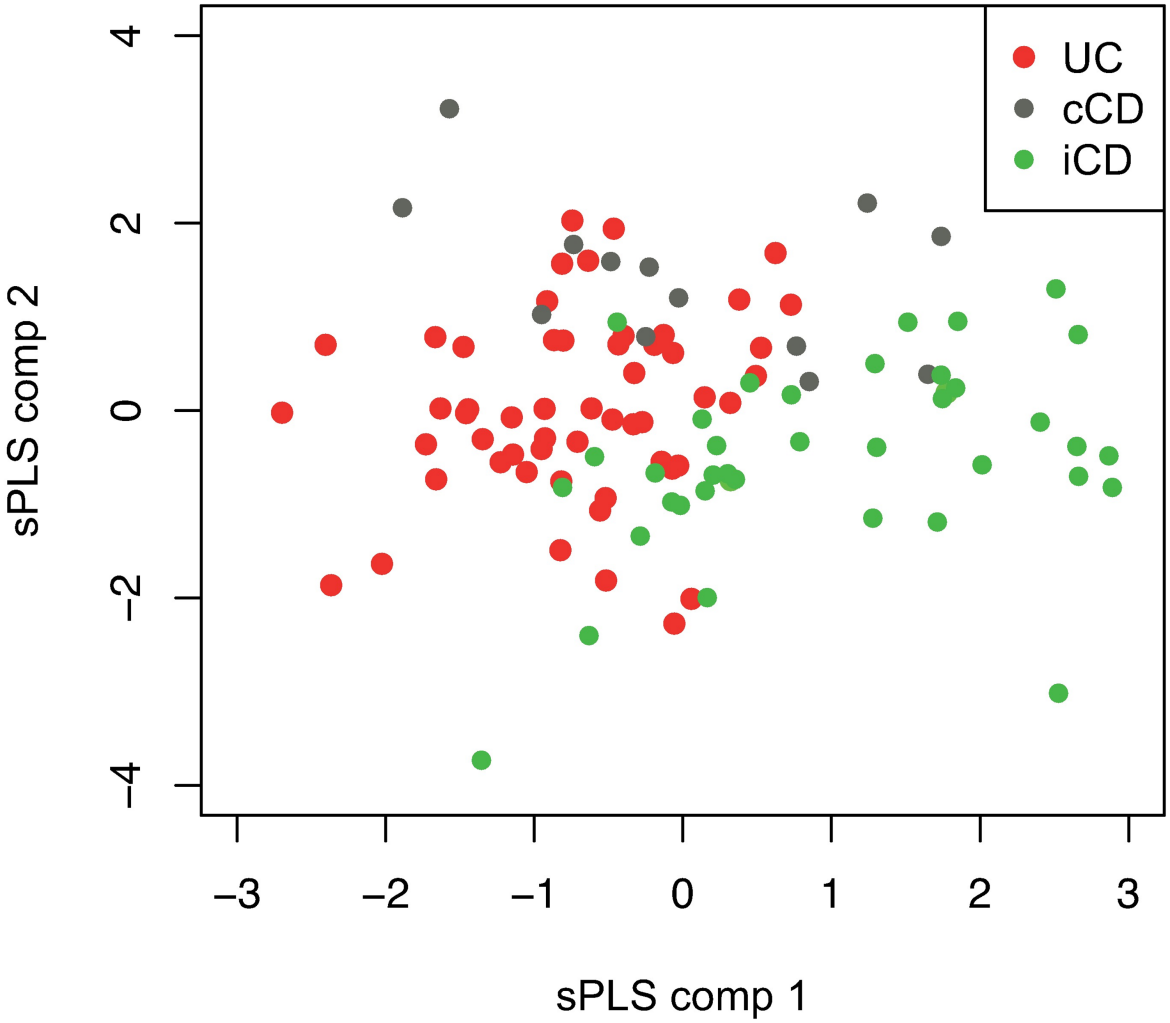
sPLS plot showing the distribution of patients with ileal CD, colonic CD, and UC in the discovery cohort, based on the 20 most important proteins from the multivariate analysis.

Proteins of importance for disease classification were then identified by discriminant sPLS analyses and cut-offs for optimal separation in each analysis were selected by the VIP score (S2 Table). All the proteins identified in univariate analysis as being significantly altered (when CD patients and UC patients were compared to HCs) were also included in the discriminant sPLS analyses. In total, 64 candidate proteins were identified as being of importance for the differentiation of CD patients from HCs. The corresponding figure for UC was 51. In addition to FGF19, discriminant sPLS analysis identified 38 additional candidate proteins when CD was compared to UC. Score plots for the first two components of the sPLS model also showed a partial differentiation of ileal/ileocolonic CD (L1/L3), colonic CD (L2), and UC samples in the discovery cohort (Fig 3). Finally, candidate proteins of importance for the differentiation of subphenotypes of CD and UC as well as for patients in clinical remission were identified by discriminant sPLS analyses (S2 Table).

Cross-validation error rates for discrimination of subgroups of individuals and corresponding resampling p-values, based on our LOO double cross-validation design, were calculated; all comparisons to HCs mounted significant p-values (Table 3).

**Table 3:** Cross-validation error rates for discrimination of subgroups of individuals and corresponding resampling p-values.

	CD *vs*. HCs	UC *vs*. HCs	UC *vs*. CD	L2 CD *vs*. L1/L3 CD	UC *vs*. L1/L3 CD	UC *vs*. L2 CD	CD Remission *vs*. HCs	UC Remission *vs*. HCs	CD Remission *vs*. UC Remission
**LOO-CV error rate**	0.12	0.19	0.41	0.29	0.28	0.35	0.14	0.22	0.32
**Resampling p-values**	<0.025	<0.025	0.1	0.175	0.85	0.075	<0.025	<0.025	0.025

LOO-CV, leave-one-out cross-validation; UC, ulcerative colitis; CD, Crohn’s disease; HCs, healthy controls; L2 CD, colonic Crohn’s disease; L1/L3 CD, ileal/ileocolonic Crohn’s disease; CD remission, Crohn’s disease patients in clinical remission; UC remission; ulcerative colitis patients in clinical remission.

### Validation of differentially regulated proteins in the replication cohort

**Fig 4:**
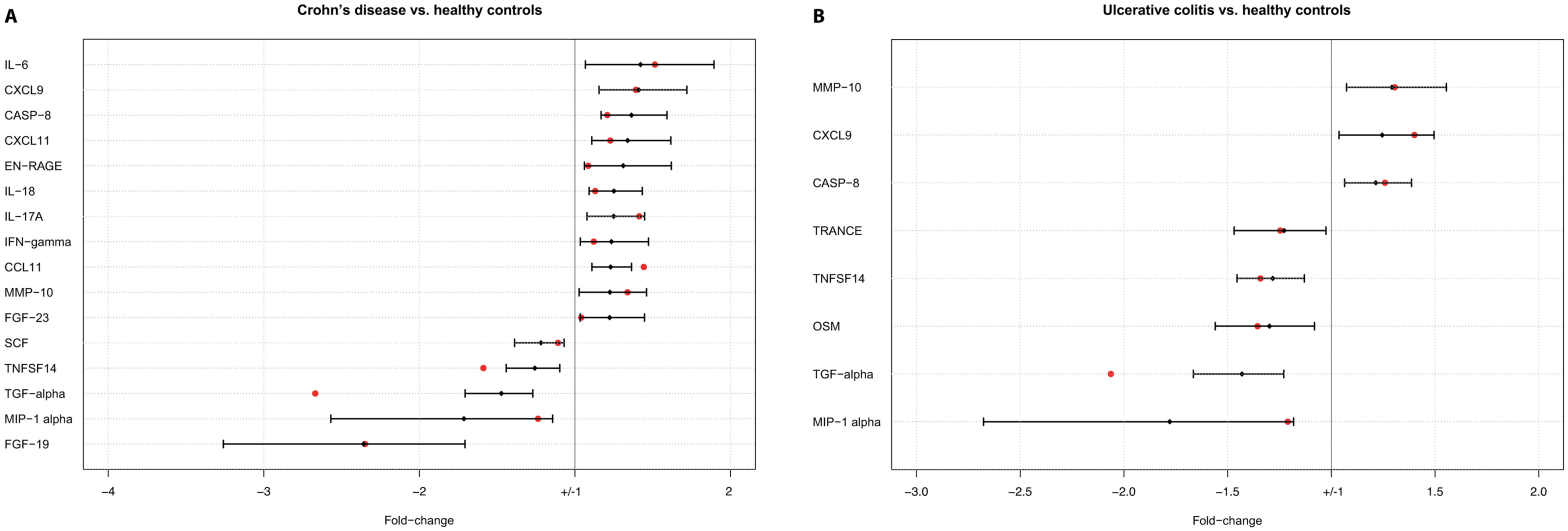
Validated proteins. Significant biomarkers (q < 0.05, fold change > 20%) from the discovery cohort that passed validation in the replication cohort shown with fold changes and 95% confidence intervals. Actual fold changes from the replication cohort are presented with a red dot in the graph. A: Proteins validated in CD versus HC samples. B: Validated proteins in UC versus HC samples.

**Fig 5:**
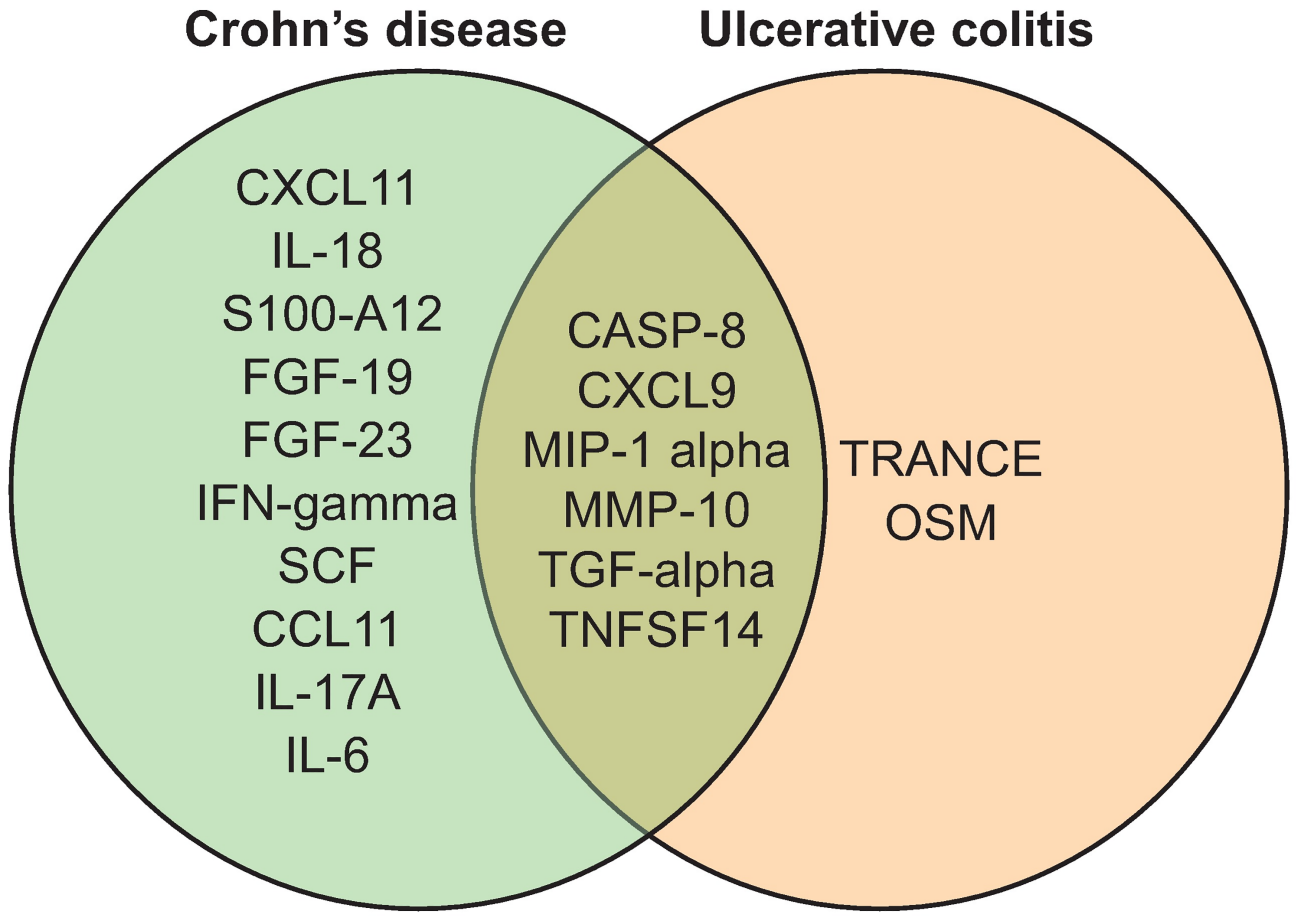
Venn diagram of validated proteins from comparison of UC patients vs. healthy controls (HCs) and CD patients vs. HCs in the replication cohort. Ten proteins were unique to CD and two were unique to UC.

The data obtained in the discovery cohort were subsequently validated in the replication cohort, consisting of 30 CD patients, 30 UC patients, and 30 HCs. The PEA of the validation samples was separate from the analysis of the discovery samples and performed at a later occasion. There were no significant differences in clinical characteristics between the discovery cohort and the replication cohort (Table 1). In total, 16 of the 17 proteins that were associated with CD―based on univariate comparisons in the discovery cohort―could be validated in the replication cohort (Fig 4A and 4B). Correspondingly, eight of the twelve proteins that were apparently associated with UC could be confirmed in the replication cohort (Fig 5). FGF-19 was also validated as being differentially regulated in CD relative to UC, and also in ileal/ileocolonic CD (L1/L3) relative to UC.

**Fig 6:**
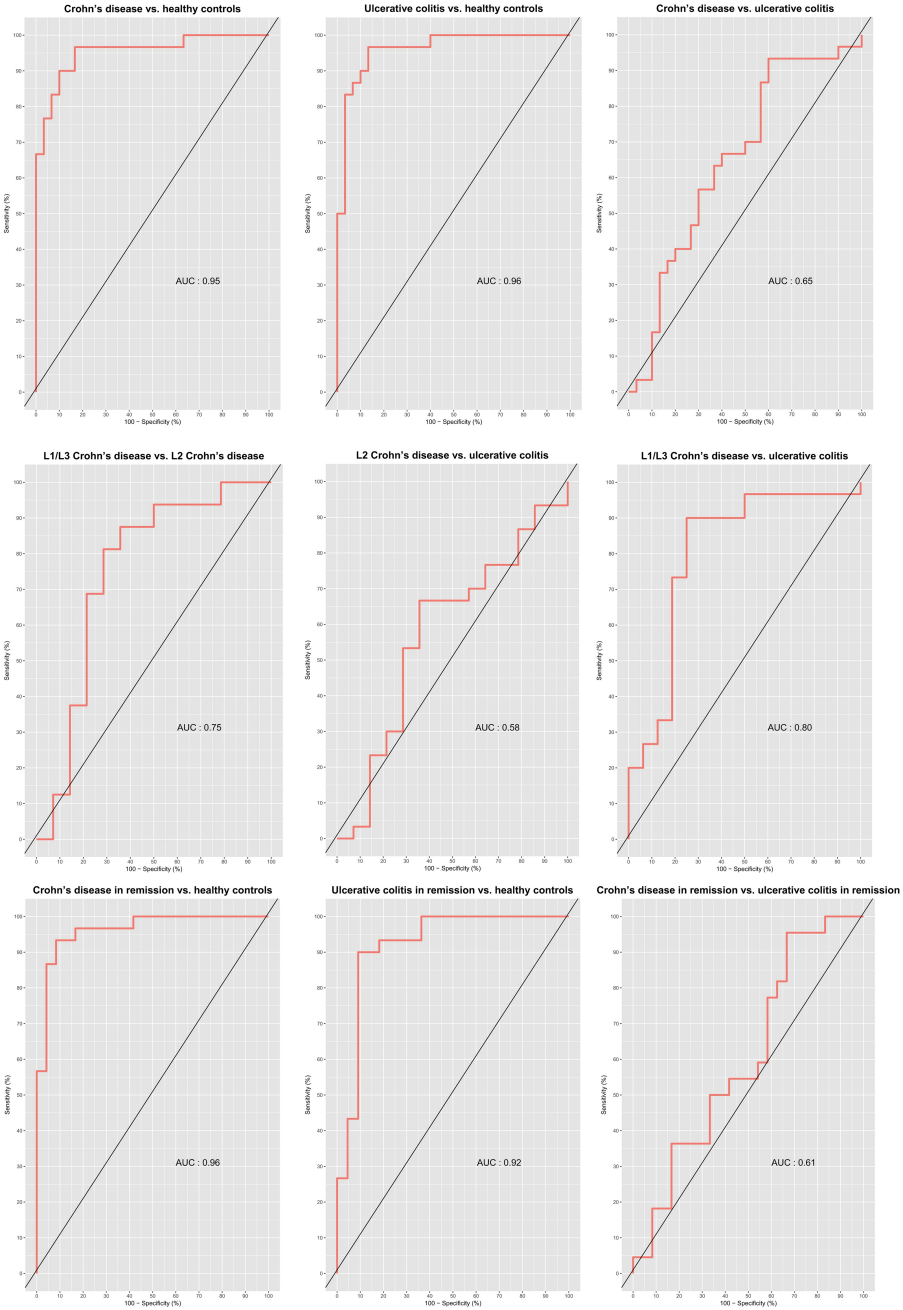
Receiver operator characteristics curves (ROC) of the sparse partial least squares models applied on the replication cohort, with area under the curves (AUCs). L2, colonic Crohn’s disease; L1/L3, ileal/ileocolonic Crohn’s disease; CD in remission, Crohn’s disease patients in clinical remission; UC in remission; ulcerative colitis patients in clinical remission.

Validation of the multivariate discrimination of subgroups in the replication cohort generated slightly higher error rates, for all models statistically significant models (Table 4). Receiver operator characteristics (ROC) curves for the different prediction models were computed (Fig 6). An area under the curve (AUC) of 0.95 was observed for CD *vs*. HCs, 0.96 for UC *vs*. HCs and 0.65 for CD *vs*. CD.

**Table 4:** Error rates of the sparse partial least-squares model for discrimination of subgroups generated from the discovery cohort, when applied to the replication cohort.

	CD *vs*. HCs	UC *vs*. HCs	UC *vs*. CD	L2 CD *vs*. L1/L3 CD	UC *vs*. L1/L3 CD	UC *vs*. L2 CD	CD Remission *vs*. HCs	UC Remission *vs*. HCs	CD Remission *vs*. UC Remission
**Error rate**	0.42	0.38	0.38	0.27	0.61	0.22	0.28	0.27	0.46

UC, ulcerative colitis; CD, Crohn’s disease; HCs, healthy controls; L2 CD, colonic Crohn’s disease; L1/L3 CD, ileal/ileocolonic Crohn’s disease; CD remission, Crohn’s disease patients in clinical remission; UC remission; ulcerative colitis patients in clinical remission.

## Discussion

There has been great progress in the characterization of the genetic landscape of IBD in recent years. However, genetic variants alone do not appear to be sufficient to cause the disease, with the possible exception of some cases of very early onset IBD. Gene expression is also influenced by epigenetic mechanisms and the transcriptome undergoes additional modification before translation into proteins. Thus, the proteome of IBD patients may reflect both genetic and environmental factors important in the pathophysiology of IBD. However, the profound difference in concentrations of different proteins in serum has hampered the exploitation of the serum proteome,[7] and previous attempts to address the IBD proteome in serum.[18–24] This drawback has been overcome by some recently introduced methods. As a proof of concept, we applied the novel proximity extension assay (PEA) technique to identify serum protein profiles of IBD using a panel of 91 proteins.

Based on univariate comparisons of CD patients and HCs in a discovery cohort and on validation in a replication cohort, 16 serum proteins were identified which differed significantly between CD patients and HCs. In the same way, eight proteins were validated in patients with UC. One of these proteins, FGF-19, was also down-regulated in CD compared with UC.

In order to identify protein profiles that are specifically associated with disease group, we applied a supervised method, that is the sPLS model. Using this supervised model, we were able to partially differentiate IBD patients from HCs based on their protein profiles. Cross-validation revealed that CD patients could be accurately discriminated from HCs in 88% of cases; the corresponding figure for UC was 81%. This accuracy of discrimination was found to be significant based on resampling analysis, although the observed accuracy dropped slightly when the model was applied to the replication cohort. The observed accuracy was supported by the ROC analyses, where the AUC was 0.95–0.96 when comparing CD and UC patients with HCs. For the comparison of subphenotypes, such as UC *vs*. colonic CD, the prediction error rates were lower and not significant. However, identification of clinically relevant biosignatures would probably benefit from inclusion of additional variables, including clinical information, and should not rely on the inflammatory protein panel only.

Several of the differentially regulated proteins that were validated in patients with CD or UC are cytokines, including IL6, IL17A, IL18, IFN-γ, eotaxin-1, CXCL9, caspase-8, and CXCL11, which have already been associated with IBD.[1, 18, 20, 25–27] Similarly, an association between the neutrophil-derived protein S100-A12 and IBD has also been reported previously. [28, 29] We were able to validate the association of S100-A12 for CD but not for UC. Since S100-A12 has been associated with active disease,[30] the non-significant change in patients with UC might be explained by the low number of patients with active disease, as 22 of the 30 patients in the replication cohort were in clinical remission. Two proteins were unique to patients with UC―TRANCE and OSM―and both were down-regulated. TRANCE (RankL) is a known activator of osteoclasts. In contrast to the data presented here, serum levels of TRANCE have previously been reported to not be different in IBD patients compared with controls.[31, 32] To our knowledge, OSM, an activator of the JAK/STAT pathway, has been studied in colonic biopsies of IBD patients only, where upregulation was reported.[33] In addition, our results also show reduction of TNFSF14 and TGF-alpha in both CD and UC. TNFSF14 has been suggested to be an important mediator of the pathogenesis in CD and in murine models neutralizing antibodies for TNFSF14 has reduced symptoms of induced colitis.[34] Conflicting results for TGF-alpha have been published before with increasing numbers of TGF-alpha containing cells in inflamed mucosa in UC, in contrast to the results of another study showing relatively lower protein signals in inflamed biopsies compared to non-inflamed biopsies in both CD and UC.[35, 36]

Intriguingly, the observed predictive power seemed to remain when stratifying for disease activity and restricting the analyses to patients in clinical remission. This observation reveals that the observed difference between IBD patients and HCs is not only seen in patients with a high systemic inflammatory burden. Hence the different phenotypes of IBD seemed to involve different inflammatory pathways that might be of interest to distinguish the different IBD entities. The univariate comparisons of CD and UC patients only identified FGF-19. FGF-19 is produced by enterocytes in the distal ileum on uptake of bile acids,[37] and a correlation with bile acid malabsorption in Crohn’s disease has recently been shown.[38] The observed decrease in serum FGF-19 was most pronounced in patients with ileal involvement, probably reflecting that 77% of the patients with ileal/ileocolonic disease had undergone surgical resection. A poor cross-evaluation error rate (0.41) and a non-significant resampling error rate were observed when trying to differentiate between CD and UC using multivariate analyses. However, the error rate seemed to improve when we stratified for location of CD and restricted the analysis to CD patients with ileal involvement (L1 and L3). These results are in line with recent genetic data from the international IBD genetic consortium (IIBDGC), where CD patients with ileal disease were reported to be more distantly related genetically to patients with UC than patients with colonic CD.[6] The model separating CD patients from UC patients also seemed to improve when stratifying for disease activity and including patients in clinical remission. The sPLS method identified CCL28 as a potential interesting marker together with FGF-19, CSF-1, IL-18, and TGF-beta-1. CCL28 has been shown to exhibit a protective function against bacteria by direct antimicrobial effect and by recruiting IgA producing leukocytes.[39, 40]

To our knowledge, most studies of the serum proteome in IBD to date, have analyzed a sparse selection of proteins. In that context, this study advances the field by introducing a novel protein signature approach. The validation process and the use of a replication cohort was a major strength of our study. On the other hand, the results were limited by the pre-selection of candidate biomarkers, since we used a predefined commercially available panel of inflammatory proteins.

A further limitation of this study is the reliance on clinical assessments of disease activity which poorly predicts mucosal inflammation.[41] Objective assessments of inflammatory activity such as endoscopic evaluation, CRP or fecal calprotectin measurements would help to address this limitation. The inflammatory activity of the healthy controls was not assed by any objective measures but subjects with acute inflammation or ongoing treatment for any inflammatory disease are not eligible as blood donors. The mixed cohort represents the many stages of IBD and the data may therefore be influenced by previous and ongoing pharmacological treatments as well as surgery. Notably, few patients were on anti-TNF therapy since patients were recruited at the outpatient clinic, mostly before the wide-spread use of biologics, and not at the infusion unit.Thus, this cohort can be used for detection of differences between established IBD and healthy controls, but it is not ideal for the purpose of diagnostic biomarker identification.

In summary, by using the novel PEA method and a panel of inflammatory proteins, we were able to identify proteins with significantly different quantities in CD patients and UC patients compared to HCs. Moreover, the protein profiles identified allowed us to partially differentiate between different subgroups of IBD patients, even when restricting the analyses to patients in clinical remission. Our work highlights the potential of the serum IBD proteome as a source for identification of future diagnostic biomarkers, but such efforts should be made in an inception cohort of treatment-naïve IBD patients, where patients with symptoms mimicking IBD are used for comparisons.

## Supporting information

S1 Table Olink ProSeek Inflammation I. Full list of measured proteins.
(DOCX)

S2 Table sPLS candidate proteins list. Full list of protein candidates with VIP values.
(XLSX)

S3 Table ProSeek Inflammation I data. Full protein panel data for the discovery and validation cohort.
(XLSX)
