# Supplementary material for: Subphenotypes of inflammatory bowel disease are characterized by specific serum protein profiles
Source: PLoS One. 2017 Oct 5;12(10):e0186142. doi: 10.1371/journal.pone.0186142 (PMC5628935; doi:10.1371/journal.pone.0186142)
Supplement: S1 Table — Full list of measured proteins. (DOCX) [file pone.0186142.s001.docx]

# S1 Table, Proteins included in the ProSeek Multiplex Inflammation I 96x96

| **Protein name** | **Abbreviation** | **Uniprot ID** |
| --- | --- | --- |
| Interleukin-8 | IL-8 | P10145 |
| Vascular endothelial growth factor A | VEGF-A | P15692 |
| Brain-derived neurotrophic factor | BDNF | P23560 |
| Monocyte chemotactic protein 3 | MCP-3 | P80098 |
| Glial cell line-derived neurotrophic factor | hGDNF | P39905 |
| CUB domain-containing protein 1 | CDCP1 | Q9H5V8 |
| Natural killer cell receptor 2B4 | CD244 | Q9BZW8 |
| Osteoprotegerin | OPG | O00300 |
| Latency-associated peptide transforming growth factor beta 1 | LAP TGF-beta-1 | P01137 |
| Urokinase-type plasminogen activator | uPA | P00749 |
| Interleukin-6 | IL-6 | P05231 |
| Interleukin-17C | IL-17C | Q9P0M4 |
| Monocyte chemotactic protein 1 | MCP-1 | P13500 |
| Interleukin-17A | IL-17A | Q16552 |
| C-X-C motif chemokine 11 | CXCL11 | O14625 |
| Axin-1 | AXIN1 | O15169 |
| TNF-related apoptosis-inducing ligand | TRAIL | P50591 |
| Interleukin-20 receptor subunit alpha | IL-20RA | Q9UHF4 |
| C-X-C motif chemokine 9 | CXCL9 | Q07325 |
| Cystatin D | CST5 | P28325 |
| Interleukin-2 receptor subunit beta | IL-2RB | P14784 |
| Interleukin-1 alpha | IL-1 alpha | P01583 |
| Oncostatin-M | OSM | P13725 |
| Interleukin-2 | IL-2 | P60568 |
| C-X-C motif chemokine 1 | CXCL1 | P09341 |
| Thymic stromal lymphopoietin | TSLP | Q969D9 |
| C-C motif chemokine 4 | CCL4 | P13236 |
| T-cell surface glycoprotein CD6 isoform | CD6 | Q8WWJ7 |
| Stem cell factor | SCF | P21583 |
| Interleukin-18 | IL-18 | Q14116 |
| Signalling lymphocytic activation molecule | SLAMF1 | Q13291 |
| [Transforming growth factor alpha](http://www.uniprot.org/uniprot/P01135#PRO_0000007753) | TGF-alpha | P01135 |
| Monocyte chemotactic protein 4 | MCP-4 | Q99616 |
| Eotaxin-1 | CCL11 | P51671 |
| Tumour necrosis factor ligand superfamily member 14 | TNFSF14 | O43557 |
| Fibroblast growth factor 23 | FGF-23 | Q9GZV9 |
| Interleukin-10 receptor subunit alpha | IL-10RA | Q13651 |
| Fibroblast growth factor 5 | FGF-5 | Q8NF90 |
| Matrix metalloproteinase-1 | MMP-1 | P03956 |
| Leukemia inhibitory factor receptor | LIF-R | P42702 |
| Fibroblast growth factor 21 | FGF-21 | Q9NSA1 |
| C-C motif chemokine 19 | CCL19 | Q99731 |
| Interleukin-15 receptor subunit alpha | IL-15RA | Q13261 |
| Interleukin-10 receptor subunit beta | IL-10RB | Q08334 |
| Interleukin-22 receptor subunit alpha-1 | IL-22 RA1 | Q8N6P7 |
| Interleukin-18 receptor 1 | IL-18R1 | Q13478 |
| Programmed cell death 1 ligand 1 | PD-L1 | Q9NZQ7 |
| Beta-nerve growth factor | Beta-NGF | P01138 |
| C-X-C motif chemokine 5 | CXCL5 | P42830 |
| TNF-related activation-induced cytokine | TRANCE | O14788 |
| Hepatocyte growth factor | HGF | P14210 |
| Interleukin-12 subunit beta | IL-12B | P29460 |
| Interleukin-24 | IL-24 | Q13007 |
| Interleukin-13 | IL-13 | P35225 |
| Artemin | ARTN | Q5T4W7 |
| Matrix metalloproteinase-10 | MMP-10 | P09238 |
| Interleukin-10 | IL-10 | P22301 |
| Tumour necrosis factor | TNF | P01375 |
| C-C motif chemokine 23 | CCL23 | P55773 |
| T-cell surface glycoprotein CD5 | CD5 | P06127 |
| Macrophage inflammatory protein 1-alpha | MIP-1 alpha | P10147 |
| Fms-related tyrosine kinase 3 ligand | Flt3L | P49771 |
| C-X-C motif chemokine 6 | CXCL6 | P80162 |
| C-X-C motif chemokine 10 | CXCL10 | P02778 |
| Eukaryotic translation initiation factor 4E-binding protein 1 | 4E-BP1 | Q13541 |
| Interleukin-20 | IL-20 | Q9NYY1 |
| SIR2-like protein 2 | SIRT2 | Q8IXJ6 |
| C-C motif chemokine 28 | CCL28 | Q9NRJ3 |
| Delta and Notch-like epidermal growth factor-related receptor | DNER | Q8NFT8 |
| Protein S100-A12 | EN-RAGE | P80511 |
| CD40L receptor | CD40 | P25942 |
| Interleukin-33 | IL-33 | O95760 |
| Interferon gamma | IFN-gamma | P01579 |
| Fibroblast growth factor 19 | FGF-19 | O95750 |
| Interleukin-4 | IL-4 | P05112 |
| Leukemia inhibitory factor | LIF | P15018 |
| Neurturin | NRTN | Q99748 |
| Monocyte chemotactic protein 2 | MCP-2 | P80075 |
| Caspase 8 | CASP-8 | Q14790 |
| C-C motif chemokine 25 | CCL25 | O15444 |
| Fractalkine | CX3CL1 | P78423 |
| Tumour necrosis factor receptor superfamily member 9 | TNFRSF9 | Q07011 |
| Neurotrophin-3 | NT-3 | P20783 |
| Tumour necrosis factor (Ligand) superfamily, member 12 | TWEAK | O43508 |
| C-C motif chemokine 20 | CCL20 | P78556 |
| Sulfotransferase 1A1 | ST1A1 | P50225 |
| STAM-binding protein | STAMPB | O95630 |
| Interleukin-5 | IL-5 | P05113 |
| Adenosine deaminase | ADA | P00813 |
| TNF-beta | TNFB | P01374 |
| Macrophage colony-stimulating factor 1 | CSF-1 | P09603 |
